# MetaboMAPS: Pathway sharing and multi-omics data visualization in metabolic context

**DOI:** 10.12688/f1000research.23427.2

**Published:** 2020-07-17

**Authors:** Julia Koblitz, Dietmar Schomburg, Meina Neumann-Schaal

**Affiliations:** 1 Department of Bioinformatics and Biochemistry, Technische Universität Braunschweig, Braunschweig, 38106, Germany; 2 Braunschweig Integrated Centre of Systems Biology, Technische Universität Braunschweig, Braunschweig, 38106, Germany; 3 Leibniz-Institut DSMZ - German Collection of Microorganisms and Cell Cultures, Braunschweig, 38124, Germany

**Keywords:** Systems Biology, Metabolic Maps, Pathways, SVG, Metabolism, Data Visualization, Omics Data

## Abstract

Metabolic pathways are an important part of systems biology research since they illustrate complex interactions between metabolites, enzymes, and regulators. Pathway maps are drawn to elucidate metabolism or to set data in a metabolic context. We present MetaboMAPS, a web-based platform to visualize numerical data on individual metabolic pathway maps. Metabolic maps can be stored, distributed and downloaded in SVG-format. MetaboMAPS was designed for users without computational background and supports pathway sharing without strict conventions. In addition to existing applications that established standards for well-studied pathways, MetaboMAPS offers a niche for individual, customized pathways beyond common knowledge, supporting ongoing research by creating publication-ready visualizations of experimental data.

## Introduction

The field of systems biology is based on the integration of data from different biological fields, e.g. transcriptomics, proteomics, metabolomics, modelling, to gain a detailed understanding of an organism. However, the data integration is still challenging to date. In particular, correlating transcriptome or proteome data to metabolome data requires careful revision based on expert knowledge of metabolic pathways and intensive manual work ( Cavill *et al.,* 2016). Therefore, easily accessible tools are needed to help during analysis and interpretation of multi-omics data. When one tries to understand metabolic changes, pathway maps are often used for guidance ( Cavill *et al.,* 2016). However, the number of pathway maps in scientific publications is large, as is the diversity. Solely the TCA cycle was drawn hundreds of times, being one of the most conserved pathways among all domains of life. However, even such conserved pathways exhibit differences among species: the gut pathogen *Clostridioides difficile* uses an incomplete TCA cycle ( Dannheim *et al.,* 2017) and some Cyanobacteria use a TCA cycle with an additional GABA shunt and a variety of anaplerotic reactions ( Will *et al.,* 2019). Conclusively, there are pathway maps that can be used for a broad range of different organisms while others are exclusive for a few species. For this reason, the overall display of pathway maps, as provided by e.g. KEGG and BRENDA, have their limitations: while pathways are widely available and immense useful for model organisms, the maps cannot provide organism- or group-specific modifications for pathways that are exclusive for small groups of organisms or are currently incompletely understood. For visualization of multi-omics data, a specific map is required both, regarding the organism and the underlying scientific question. Here we present MetaboMAPS ( Koblitz, 2020), a novel web-based tool that on one hand, serves as a platform to share metabolic pathway maps in an organism-dependent manner. On the other hand, MetaboMAPS assists during interpretation of metabolism-associated data by visualizing experimental data sets on pathway maps.

## Methods

### Implementation

PHP is used to access an internal SQL database and to handle file and user management. In addition, a user-friendly web interface is integrated to handle pathway exploration and user interactions. The pathways can be uploaded, stored and downloaded in SVG format. SVG manipulation, including zoom, editing, and plotting of data, is done with the JavaScript Library D3. Hosting, infrastructure maintenance, and issue tracking is provided by the enzyme database BRENDA.

### Operation

MetaboMAPS ( Koblitz, 2020) can be accessed with every modern browser. Log-in is required for upload, editing, and sharing of pathways, but not for exploring pathways, data visualization and downloads.

## Results

### Sharing metabolic pathways

MetaboMAPS ( Koblitz, 2020) is a platform where users can upload individual metabolic pathways and release them for the scientific community. In this process, the pathway gets a unique accession number for reference in publications. Furthermore, the user can link pathways to publications. If the pathway map includes unpublished information, it can be uploaded in confidential mode. In this way, the pathway can be shared with specified colleagues and used for data visualization, but is not available for the general public. Pathways can be found by searching category, name, assigned identifier (e.g. EC number, locus tag), or accession. A unique feature of MetaboMAPS is that uploaded maps must not follow strict conventions as other tools require. The style, detail level and content of the maps is according to the scientist’s needs, and since the maps can be downloaded and modified, they can also be adjusted by other users. Pathway rating and the possibility to add comments increase the quality of uploaded pathways via community contributions. In this way, MetaboMAPS does not compete with but complements curated, comprehensive maps that are already well established. It offers a niche for tentative, novel or incomplete pathways to support ongoing research beyond common knowledge. Since MetaboMAPS creates reproducible, customizable visualizations of high quality, it is suitable to generate publication-ready figures with little effort.

Each pathway is associated with one or more organisms. In fact, it is possible to add the same pathway to hundreds of different organisms. An organism overview shows all pathway maps that are associated to a selected organism. On the other hand, the pathway overview page displays all background information, such as a list of authors, the pathway description, links to publications, and all organisms that are associated to this pathway. The pathways and information are also easily accessible on mobile devices.

Users can upload their own metabolic pathways in SVG format. We chose this particular format because it can be displayed in every modern browser, can be easily manipulated, is completely scalable, and of small file size. Additionally, SVG-files can be exported from every program that users eventually use to draw a metabolic pathway (e.g. Inkscape, Adobe Illustrator, Microsoft Powerpoint, LibreOffice Impress) and users can continue to work with their preferred software.

### Multi-omics data visualization

**Figure 1.**
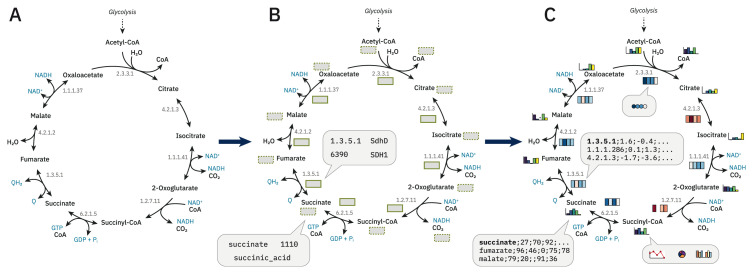
The process of data visualization on MetaboMAPS. ( **A**) Upload the metabolic pathway in SVG format. Alternatively, you can use an existing pathway. ( **B**) Draw plot boxes for metabolite (dashed border) and reaction (solid border) associated data. Assign identifiers to each plot box (e.g. EC numbers, locus tags, GI, metabolite synonyms, database IDs). ( **C**) Load your own data set (as CSV file) and visualize reaction and metabolite dependent data simultaneously.

A unique and highly useful feature of MetaboMAPS is the possibility to visualize experimental data on metabolic pathways. Suitable data sets include but are not limited to transcriptomic, proteomic, metabolomic studies, flux distributions, 13C-flux measurements and others. The process for sharing pathway maps and using them for visualization is shown in Figure 1. The first step is the upload of an existing metabolic pathway in SVG format ( Figure 1A). Afterwards, the user can add further information and assign the pathway to a pathway category. In the second step, an intuitive online editor is used to draw plot boxes ( Figure 1B), which define the positions where the experimental data should be visualized. Each plot box can be assigned to one or more identifiers, either organism-specific (e.g. locus tags, GIs) or general (e.g. EC numbers, metabolite names). Data from the BKMS ( Lang *et al.,* 2011) and BRENDA ( Jeske *et al.,* 2019) databases are used to provide auto-completion of metabolites and enzymes, synonym matching, and cross-linking identifiers to other databases, e.g. BRENDA, KEGG, and MetaCyc. The identifier connects a row in the uploaded data set to a specific plot box. In the third step, any type of numerical data can be loaded in the browser and is visualized in the respective plot box ( Figure 1C). Data must be in CSV-format, containing the identifiers that connect the data to plot boxes in the first row. Different types of visualization, like colour scales, a number of plot types (e.g. bar charts, line charts, heat maps), and other visual settings offer a high degree of customization. In the end, the pathway including the data visualization as well as legends can be downloaded in SVG or PNG-format.

## Discussion

Among the resources for biological pathway maps, the KEGG pathways (> 500 pathways; Kanehisa *et al.*, 2019), MetaCyc (> 3800 pathways; Caspi *et al.*, 2018), and WikiPathways (>2800 pathways; Slenter *et al.*, 2018) are most considerable, having thousands of users per month and offering a large number of pathway maps. These tools differ in their application and are appropriate for different use cases. KEGG offers revised maps of high quality that can be used for visualization purposes mainly by R packages ( Luo *et al.*, 2017). MetaCyc has a large number of organism-specific pathways that are popular among biochemists, but are barely used for data integration. In contrast to the other tools, WikiPathways is a community-driven approach, that requires a little effort from the users by relying on PathVisio for pathway creation. PathVisio ensures that pathways are created in compliance with established standards and can also be used to integrate experimental data.

At this moment, MetaboMAPS is a relatively small resource, but it is intended to grow with the community. The main difference to other approaches is the freedom in pathway creation and the data visualization without relying on specific external software, particular data formats or steep learning curves. MetaboMAPS allows specific, indivualized or yet incompletely understood pathways and link them to the demands on data visualization. This complements the major pathway resources available. Linking the pathways to publications will also allow re-use of the pathway. MetaboMAPS addresses biologists with nominal bioinformatical knowledge and researchers that want to share published pathway maps without much effort.

## Conclusion

In summary, MetaboMAPS ( Koblitz, 2020) is a platform for sharing metabolic pathway maps and visualizing data in a metabolic context. It encourages scientists to share individual pathway maps without strict conventions and offers customizable and reproducible visualizations of experimental data. It will grow in collaboration with the community and by further development by the BRENDA team.

## Data availability

All data underlying the results are available as part of the article and no additional source data are required.

### Software availability

**Software available from:** https://metabomaps.brenda-enzymes.org.

**Source code available from:** https://github.com/JuliaHelmecke/MetaboMAPS.

**Archived source code at time of publication:** https://doi.org/10.5281/zenodo.3742817 ( Koblitz, 2020).

**License:** GNU General Public License v3.0 or later.
