# Supplementary material for: MetaboMAPS: Pathway sharing and multi-omics data visualization in metabolic context
Source: F1000Res. 2020 Jul 17;9:288. Originally published 2020 Apr 24. [Version 2] doi: 10.12688/f1000research.23427.2 (PMC7383707; doi:10.12688/f1000research.23427.2)
Supplement: Peer review history

# Peer review: Reviewer response for version 2

**DOI:** 10.5256/f1000research.27654.r67345

**Published:** 2020-07-24

**Authors:** Bianca H. Habermann

**Affiliations:** 1 Aix-Marseille University, CNRS, IBDM UMR 7288, Marseille, France

The authors have addressed my concerns adequately.

Are the conclusions about the tool and its performance adequately supported by the findings presented in the article?

Yes

Is the rationale for developing the new software tool clearly explained?

Yes

Is the description of the software tool technically sound?

Yes

Are sufficient details of the code, methods and analysis (if applicable) provided to allow replication of the software development and its use by others?

Yes

Is sufficient information provided to allow interpretation of the expected output datasets and any results generated using the tool?

Yes

Reviewer Expertise:

Computational Biology, Systems Biology, Data Integration, Evolutionary Biology, Mitochondria

I confirm that I have read this submission and believe that I have an appropriate level of expertise to confirm that it is of an acceptable scientific standard.

# Peer review: Reviewer response for version 1

**DOI:** 10.5256/f1000research.25857.r63432

**Published:** 2020-06-10

**Authors:** Bianca H. Habermann

**Affiliations:** 1 Aix-Marseille University, CNRS, IBDM UMR 7288, Marseille, France

MetaboMAPS is an interesting small application for making available and displaying numerical data on non-standard pathways. It can be seen as complementing the well-known and standard pathway resources, such as KEGG and others. The advancement of MetaboMAPS is the possibility to store and present incomplete or so far not described pathways. Moreover, it has a nice visualization toolbox to display numerical data on the pathways.

 There are some points that should be addressed: 

1. The way the introduction is formulated currently indicates that well-established pathway resources are ignorant concerning species-specific differences of pathways. This is not true. KEGG for instance offers a rich selection of organisms and indicates that genes are missing/added to the classical pathway. It is true however when it comes to the overall display of the pathway map, which is – I think – what the authors want to express here. They should reformulate this to reflect what they mean to say properly.
2. I do not fully understand, why it is necessary to point out the disadvantages of printed pathway maps (in a book, citation Michal & Schomburg) – which by default cannot be used for any form of computerized work? 
3. Sparse information is given – also on the website – of how to upload user-provided pathways. For instance, it seems necessary to add an organism, before adding a pathway. This is very un-intuitively presented at the website and should be improved.
4. The SVG format offers many advantages. However, it is not so easy to e.g. build on an existing pathway from other resources, as they often do not offer download in SVG format. Do the authors have any thoughts on how the interoperability with existing pathway resources could be improved, e.g. by allowing more and different formats for upload?
5. Should the resource become more widely used for pathways that are incomplete, it will become a problem that users don’t stick to the same nomenclature. How do the authors think about consolidating novel pathways coming from multiple users that illustrate the same biological pathway, yet under a different name?
6. Whether or not it is an advantage to offer such great flexibility with respect to the pathway map is questionable. Often, users don’t sick to official gene symbols, but use synonyms or other, officially not recognized gene names. The authors should comment on how they could address this issue of unconventional gene names or rarely used synonyms.

Are the conclusions about the tool and its performance adequately supported by the findings presented in the article?

Yes

Is the rationale for developing the new software tool clearly explained?

Yes

Is the description of the software tool technically sound?

Yes

Are sufficient details of the code, methods and analysis (if applicable) provided to allow replication of the software development and its use by others?

Yes

Is sufficient information provided to allow interpretation of the expected output datasets and any results generated using the tool?

Yes

Reviewer Expertise:

Computational Biology, Systems Biology, Data Integration

I confirm that I have read this submission and believe that I have an appropriate level of expertise to confirm that it is of an acceptable scientific standard, however I have significant reservations, as outlined above.

# Response

**Published:** 2020-06-23

**Authors:** Julia Koblitz

**Affiliations:** Braunschweig University of Technology, Germany

We highly appreciate your feedback regarding MetaboMAPS and the quality of our paper. We uploaded a new version of the latter to address your concerns.

 Point 1 and 2: We changed to the manuscript accordingly.

 Point 3: Thank you for your helpful feedback regarding the upload of pathway maps. We reimplemented the upload procedure to be more intuitive and user friendly. Basically, the user can now define which organisms are associated after uploading a pathway. Furthermore, we added a submenu to the navigation bar.

 Point 4: Interoperability is an important topic. The SVG format was our favourite choice because it can be exported from nearly every tool that users eventually use to draw their pathways. Additionally, there exists a number of conversion tools to convert nearly every vector-based format to SVG (we provide How-Tos on the website). Also existing platforms often provide export to SVG, e.g. we tried to add pathways exported from WikiPathways and BRENDA and it worked perfectly fine. However, we are open to include other data formats (e.g. KGML) in the future. Therefore, we would like to rely on user surveys once the tool has a larger community.

 Point 5: Regarding concerns about pathway nomenclature, we agree that this will be an issue in the future. At this point, we group pathways roughly using 19 pathway categories. Pathways can furthermore be found using identifiers such as EC numbers or metabolites using the search function. However, with increasing numbers of pathways, we will consider implementing a system based on pathway ontologies (e.g. https://www.ebi.ac.uk/ols/ontologies/pw) at a later time point.

 Point 6: We agree that flexibility is associated with high variability. For this reason, we implemented a system underlying the identifiers that gives the most possible consistency and connectivity, but maintains the flexibility: among inofficial gene names and locus tags, EC numbers are used for reactions and linked to the BRENDA enzyme database. Furthermore, a synonym database is used to match all known metabolite synonyms and connect them to other databases, i.e. BRENDA, KEGG, MetaCyc, and Sabio-RK.

# Peer review: Reviewer response for version 1

**DOI:** 10.5256/f1000research.25857.r63026

**Published:** 2020-05-28

**Authors:** Rachel Cavill

**Affiliations:** 1 Department of Data Science and Knowledge Engineering, Maastricht University, Maastricht, The Netherlands

The authors, through this short descriptive paper, present MetaboMaps, a new tool for uploading pathways and overlaying data from multiple omics. The pathways are visualised clearly with multiple options for the type of overlays available.  

 The program is open source and publicly available, making it easy for others to access and examine as needed.

 This paper would be strengthened by including a better review of other platforms/tools which do similar or related tasks. In particular, the authors should look at wikipathways, and the combination of wikipathways with pathvisio or cytoscape, and explore how their approach differs from what is currently available through these tools. By highlighting the differences and similarities between approaches it will make it easier for potential users to evaluate the platform and select the best tool for their datasets.

Are the conclusions about the tool and its performance adequately supported by the findings presented in the article?

Yes

Is the rationale for developing the new software tool clearly explained?

Partly

Is the description of the software tool technically sound?

Yes

Are sufficient details of the code, methods and analysis (if applicable) provided to allow replication of the software development and its use by others?

Yes

Is sufficient information provided to allow interpretation of the expected output datasets and any results generated using the tool?

Yes

Reviewer Expertise:

multi-omics integration, pathway analysis, omics data visualisation.

I confirm that I have read this submission and believe that I have an appropriate level of expertise to confirm that it is of an acceptable scientific standard.

# Response

**Published:** 2020-06-23

**Authors:** Julia Koblitz

**Affiliations:** Braunschweig University of Technology, Germany

Thank you for your feedback regarding our manuscript. We uploaded a new version of our paper and included a review on other resources as suggested.
